# Global burden of subarachnoid hemorrhage attributable to ambient PM_2.5_ in low-resource regions (1990–2050)

**DOI:** 10.3389/fpubh.2025.1652872

**Published:** 2025-09-23

**Authors:** Erman Wu, Tong Tang, Riqing Su, Yandong Li, Maimaitili Mijiti, Gaocai Zhang, Minghao Lian, Yongtao Zhang, Chang Du, Guohua Zhu, Dangmurenjiafu Geng

**Affiliations:** ^1^Department of Neurosurgery, The First Affiliated Hospital of Xinjiang Medical University, Urumqi, China; ^2^Department of Computer Science and Information Technologies, University of A Coruña, A Coruña, Spain; ^3^School of Life Science, South China Normal University, Guangzhou, China

**Keywords:** PM2.5, subarachnoid hemorrhage, disease burden, estimated annual percentage changes, Bayesian age-period-cohort modeling

## Abstract

**Background:**

Subarachnoid hemorrhage (SAH) is increasingly recognized as a PM_2.5_-linked neurological emergency, yet global spatiotemporal burden evidence across socioeconomic, demographic, and geographic subgroups remains scarce, impeding tarsgeted prevention. This study quantifies current burden, trends, and future SAH projections attributable to PM_2.5_ using the latest data.

**Methods:**

Using data from the Global Burden of Disease Study 2021, we analyzed deaths and disability-adjusted life years (DALYs) from SAH attributable to ambient PM_2.5_ pollution (1990–2021) across 204 countries/territories, stratified by age, sex, region, and Socio-demographic Index (SDI). Temporal trends were quantified using estimated annual percentage changes (EAPCs), and Bayesian age-period-cohort modeling projected disease burden through 2050.

**Results:**

Between 1990 and 2021, global age-standardized mortality (ASMR) and DALY rates (ASDR) for PM_2.5_-related SAH declined by 36% (0.99 to 0.63 per 100,000) and 34% (27.42 to 17.96 per 100,000), respectively. However, absolute deaths surged 40% (38,130 to 53,562), driven by aging populations and demographic shifts. Burden disparities were stark: Middle SDI regions had the highest ASMR (1.07, 95% UI, 0.68–1.43) and ASDR (27.42, 95% UI: 17.96–35.65), while high SDI regions achieved the steepest declines (−67% ASMR). South Asia (+246% deaths) and Southeast Asia (+147% deaths) experienced the most rapid mortality growth, contrasting with East Asia’s high absolute burden (229,553 deaths in 2021). Males faced higher risks (ASMR: 0.72, 95% UI: 0.48–0.99) compared with females (0.55, 95% UI: 0.36–0.75). In South Asia, the female mortality share was rising from 31 to 41%. Mongolia had the highest national burden [2.49 (95% UI, 1.23–3.82) and ASDR of 61.92 (95% UI, 30.6–93.24)], while Central Asia and Southern Sub-Saharan Africa exhibited worsening trends. Projections indicate a resurgence in ASMR and ASDR by 2050, disproportionately impacting low-middle SDI regions.

**Conclusion:**

Despite declining age-standardized rates, a 40% surge in absolute PM_2.5_-attributable SAH deaths over three decades, due to aging populations and regional inequalities (e.g., South Asia +246% deaths, Middle SDI highest ASMR), demands urgent air-quality and healthcare policies for high-growth Asian and African regions and vulnerable low-middle SDI populations to curb projected 2050 increases.

## Introduction

Subarachnoid hemorrhage (SAH), a devastating form of hemorrhagic stroke caused by bleeding into the subarachnoid space, represents a critical global health challenge with substantial regional variations in disease burden (1, 2). SAH, a devastating form of hemorrhagic stroke caused by bleeding into the subarachnoid space, accounts for 5–10% of all stroke cases globally and is associated with high mortality rates, particularly in regions such as Central Asia and Eastern Europe (3). According to the Global Burden of Disease Study 2021, while the crude incidence of SAH increased by 37.09% from 1990 to 2021, the age-standardized incidence rates decreased significantly (EAPC: -1.52; 95% UI -1.66 to −1.37) (4). International comparisons reveal striking disparities, with the highest age-standardized incidence rates observed in the high-income Asia Pacific region at 14.09 per 100,000 population, while regions such as East Asia experienced substantial decreases with an estimated annual percentage change of −3.60 (1, 4). The global incidence declined from 10.2 per 100,000 person-years in 1980 to 6.1 in 2010, with notable variations according to region, blood pressure levels, and smoking prevalence (5). These epidemiological disparities between countries and regions highlight the necessity for comprehensive global research initiatives to understand underlying risk factors, optimize prevention strategies, and improve treatment protocols across diverse populations and healthcare systems.

The emerging evidence for environmental pollutants as modifiable risk factors for SAH has garnered increasing attention in recent epidemiological research, with multiple studies demonstrating significant associations between air pollution exposure and cerebrovascular events (6, 7). PM_2.5_, a pollutant capable of penetrating the bloodstream and inducing systemic inflammation and oxidative stress, has been linked to cerebrovascular damage and aneurysm rupture (8–10). Recent studies suggest that even short-term exposure to PM_2.5_ increments as low as 1 μg/m^3^ may elevate SAH risk by 1.7%, underscoring its public health significance (11). A landmark study in South Korea revealed gender-specific associations, showing that districts with higher interquartile range concentrations of NO₂ (12.2 ppb), SO₂ (1.41 ppb), and PM₁₀ (9.4 μg/m^3^) had 1.06, 1.06, and 1.05-fold higher mortality rates from SAH in females, respectively, while no significant associations were observed in male (12). This finding is corroborated by research indicating that air pollution effects on stroke mortality demonstrate stronger associations in women than men, potentially due to differential susceptibility mechanisms (13). Additional research from Seoul demonstrated that among meteorological and pollutant variables, ozone was independently associated with subarachnoid hemorrhage occurrence (14), while global burden studies indicate that air pollution-related stroke deaths, including SAH, reached 1,989,686 deaths globally in 2021 (15). These findings collectively emphasize the critical importance of environmental pollution as a modifiable risk factor for SAH and highlight the urgent need for public health interventions targeting air quality improvement as a strategy for cerebrovascular disease prevention.

Despite growing recognition of PM_2.5_’s role in SAH, critical knowledge gaps persist. First, the burden of PM_2.5_-attributable SAH across diverse geographic and demographic subgroups remains poorly quantified. For instance, sex-specific susceptibility (e.g., heightened male vulnerability potentially tied to vascular physiology) (16), and age-related disparities (e.g., increased risks in children and the older adults due to developmental or immunosenescence factors) (17, 18) warrant systematic investigation. Second, socioeconomic inequities, as reflected by the Socio-Demographic Index (SDI), may exacerbate PM_2.5_-related SAH burdens in low-resource settings with limited air quality regulations (19–21). Third, while prior ecological studies have examined regional PM_2.5_-SAH associations (22, 23), no global analysis has projected long-term trends to inform policy planning. Therefore, this study uses the latest GBD 2021 data to assess the global disease burden (mortality and DALYs) of SAH caused by PM2.5 (both APMP and HAP), analyze the trends from 1990 to 2021 with the EAPC to better understand the complex patterns, forecast the future burden of PM2.5-attributable SAH until 2050, and examine these burdens and trends across different countries, regions, genders, and age groups.

## Methods

### Study data

The GBD 2021 delivers through evaluation of disease, injury, and risk factor burden across 204 countries and territories, encompassing 88 risk factors (24, 25). This research offers findings on incidence, prevalence, mortality, DALYs, YLDs, and YLL for 371 diseases in 204 countries and regions. It uses data on 88 risk factors from 1990–2021, along with associated uncertainty intervals (UIs). Study data were sourced from the GBD 2021, accessible through https://ghdx.healthdata.org/gbd-2021. The risks are organized into a four-tier hierarchy: Level 1 encompasses environmental, occupational, behavioral, and metabolic risks; Level 2 details include 20 broader categories such as air pollution and high BMI; Level 3 encompasses more nuanced risks, including particulate matter pollution and child growth failure, representing some of the most detailed categorizations. Further refinement occurs at Level 4, which breaks down risks from Level 3 into even more specific classification, such as ambient particulate matter pollution and child stunning. The four-level risk hierarchy is based on the well-established comparative risk assessment (CRA) framework developed by the Global Burden of Disease (GBD) Study, which has been widely adopted as the international standard for risk factor quantification (26, 27). Data processing followed standardized GBD protocols (24, 25). Input data underwent: Quality grading (0–5 stars based on completeness, diagnostic specificity, and representativeness), Bias adjustment via spatial–temporal meta-regression, Ensemble modeling integrating 43 cause-of-death models. This framework replaces conventional systematic review tools by directly quantifying uncertainty from source heterogeneity.

The Socio-demographic Index (SDI) measures development by amalgamating income, education, and fertility data, classifying regions into five development stages from Low to High SDI, reflecting population wealth and education levels. Specifically, the SDI ranges are as follows: Low SDI from 0 to 0.4658, low-middle SDI from 0.4658 to 0.6188, Middle SDI from 0.6188 to 0.7120, High-middle SDI from 0.7120 to 0.8103, and High SDI from 0.8103 to 1.

In the 10th edition of the International Classification of Disease (ICD-10), subarachnoid hemorrhage is classified under codes I60-I60.9, I62.0, I67.0-I67.1, and I69.0. The ICD-10 assigns the code 430–430.9 to subarachnoid hemorrhage.

DALY (Disability-Adjusted Life Years): A summary measure of population health that quantifies the burden of disease by combining years of life lost due to premature mortality and years lived with disability, weighted by the severity of the disability (28). ASMR (Age-Standardized Mortality Rate): A mortality rate that has been adjusted to account for differences in age structure between populations, allowing for valid comparisons across different populations and time periods (29). ASDR (Age-Specific Death Rate): The number of deaths in a specific age group per 100,000 population in that same age group during a given time period (30).

### Definition of ambient PM_2.5_

Ambient particulate matter pollution refers to the annual average concentration of PM_2.5_, particles smaller than 2.5 micrometers in diameter, in the air, weighted by population exposure. This estimation integrates data from various sources, such as satellite aerosol observations, ground-level air quality monitors, chemical transport models, demographic data, and land-use information. For the GBD 2021, the dataset was expanded with newer ground monitor readings from both pre-existing and newly added sites. Additional contributions to the database came from sources such as the European Environment Agency, the United States Environmental Protection Agency, and the OpenAQ initiative (31).

### Statistical analysis

The Estimated Annual Percentage Change (EAPC) serves as a pivotal indicator for monitoring the progression of Age-Standardized Rate (ASR) across time. It is determined within a regression framework defined as y = *α* + *β*x + *ε*, where y corresponds to the annual rate change to per 100,000 individuals, α represents the intercept, β represents the slope, x is the calendar year, and ε is the error term. The EAPC calculation is based on the formula:


$$\text{EAPC}=100^{*}\;(\exp(\beta )–1)$$

With the 95% confidence interval (CI) extracted directly from the linear regression model parameters. Statistical significance is established for two-sided *p*-values below 0.05.

In parallel, the Pearson correlation coefficient was applied to evaluate the relationship between ASR and the SDI, with significance indicated by p-values less than 0.001. These analyses were conducted using R software, version 4.4.1.

### Projection analysis

The Bayesian Age-Period-Cohort (BAPC) R package is a statistical tool designed for projecting future disease burdens using a Bayesian framework (29). We employed age-specific population data spanning from 1990 to 2021, along with projections for 2022 to 2050, to assess trends in mortality and DALYs among populations. For our analysis, we adhered to the standard parameters provided with the BAPC packages, leveraging its default settings to effectively model.

## Results

### Global PM_2.5_-attributable SAH burden by regions from 1990 to 2021

**Figure 1:**
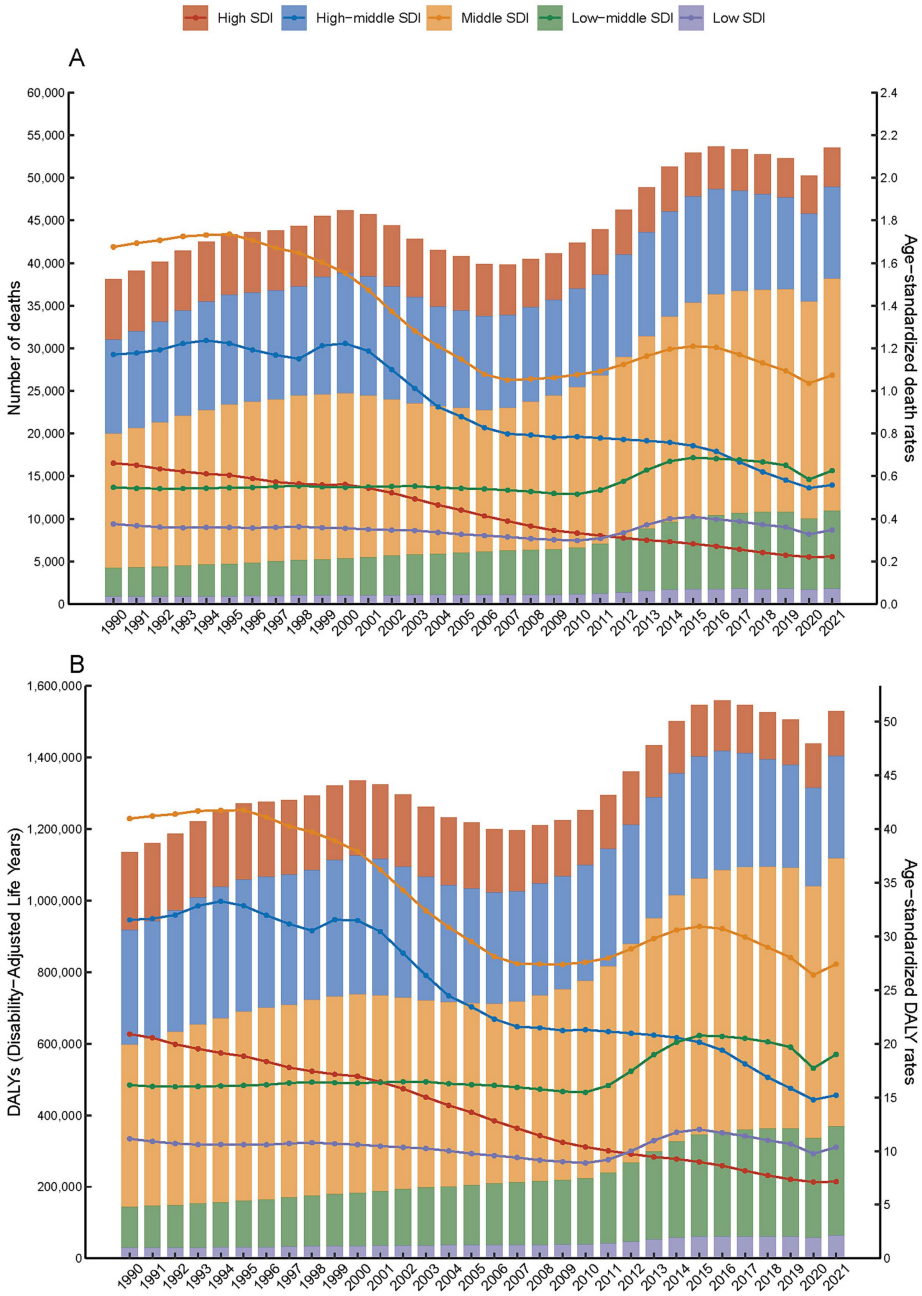
Number and age-specific rates of disease burden (**A**. number of death and age-standardized death rates; **B**. DALYs and age-standardized DALYs rates) for subarachnoid hemorrhage attributed to PM2.5 across five SDI quintiles, 1990–2021.

The trends in age-standardized mortality rates (ASMR) and age-standardized DALY rates (ASDR) for SAH attributed to ambient PM_2.5_ have shown a downward trend from 1990 to 2021, except for regions with low-middle SDI (Figure 1 and Supplementary Figure S1). Despite a decline in ASMR from 0.99 (95% UI, 0.60–1.50) in 1990 to 0.63 (95% UI, 0.43–0.82) in 2021, the death toll rose from 38,129.84 (95% UI, 23,179.25–57695.62) in 1990 to 53,561.65 (95% UI, 36,516.80-69,717.63) in 2021. Within the spectrum of SDI regions, middle SDI exhibited the highest ASMR and ASDR at 1.07 and 27.42, respectively. In addition, high SDI regions experienced the most pronounced decline in ASMR and ASDR, from 0.66 and 20.9 in 1990 to 0.22 and 7.14 in 2021. Turning to gender differences, ASMR for SAH due to PM_2.5_ was higher in male at 0.72 compared to female at 0.55 in 2021 (Tables 1, 2).

**Table 1:** Trends in PM_2.5_-Attributable subarachnoid hemorrhage mortality from 1990 to 2021 by geographic region.

Characteristics	1990		2021		1990–2021
	The number of deaths (95% UI)	Age-standardized death rates (95% UI)	The number of deaths (95% UI)	Age-standardized death rates (95% UI)	EAPC of age-standardized death rates (95% CI)
Global	38129.84 (23179.25–57695.62)	0.99 (0.6–1.5)	53561.65 (36516.8–69717.63)	0.63 (0.43–0.82)	−1.73 (−1.98--1.48)
Female	18263.68 (11080.11–27633.28)	0.87 (0.53–1.32)	25512.76 (16647.26–34523.29)	0.55 (0.36–0.75)	−1.83 (−2.09--1.57)
Male	19866.15 (10374.06–32984.81)	1.13 (0.57–1.89)	28048.9 (18640.12–38656.26)	0.72 (0.48–0.99)	−1.66 (−1.92--1.4)
Low SDI	853.13 (323.21–1670.96)	0.38 (0.14–0.75)	1810.62 (815.05–3563.04)	0.35 (0.16–0.69)	−0.03 (−0.34–0.27)
Low-middle SDI	3364.78 (1692.98–5863.75)	0.55 (0.27–0.97)	9129.25 (4917.95–14337.5)	0.63 (0.34–0.99)	0.64 (0.38–0.89)
Middle SDI	15745.67 (7707.4–26582.97)	1.68 (0.78–2.87)	27225.42 (17331.6–36189.65)	1.07 (0.68–1.43)	−1.8 (−2.16--1.44)
High-middle SDI	11035.37 (6768.97–16841.99)	1.17 (0.72–1.79)	10782.99 (7834.57–14951.13)	0.56 (0.4–0.77)	−2.68 (−2.96--2.4)
High SDI	7096.72 (4349.21–10,500)	0.66 (0.4–0.98)	4579.75 (3079.52–6324.39)	0.22 (0.15–0.3)	−3.8 (−4.01--3.58)
Australasia	37.85 (1.31–107.73)	0.17 (0.01–0.47)	66 (39.03–100.57)	0.12 (0.07–0.19)	−1.45 (−1.95--0.94)
Oceania	13.11 (3.25–33.27)	0.45 (0.12–1.16)	31.82 (9.85–71.45)	0.42 (0.13–0.94)	−0.44 (−0.65--0.24)
East Asia	17599.29 (6747.62–33368.15)	2.47 (0.92–4.69)	22952.99 (14079.23–31507.05)	1.13 (0.69–1.54)	−2.93 (−3.45--2.41)
Central Asia	258.35 (101.52–498.76)	0.56 (0.22–1.08)	677.3 (441.5–898.15)	0.88 (0.57–1.17)	2.31 (1.7–2.94)
South Asia	3201.81 (1262.05–6673.73)	0.54 (0.21–1.15)	11091.32 (6047.88–17680.03)	0.73 (0.4–1.18)	1.22 (0.87–1.57)
Southeast Asia	2149.93 (926.12–4112.56)	0.88 (0.37–1.69)	5309.19 (3332.28–7740.99)	0.86 (0.53–1.28)	−0.53 (−0.78--0.28)
High-income Asia Pacific	2017.2 (575.73–4095.88)	1 (0.29–2.04)	1846.9 (1045.07–2782.59)	0.41 (0.24–0.61)	−3.13 (−3.41--2.86)
Eastern Europe	2775.65 (1328.7–4386.38)	1.07 (0.51–1.7)	1522.11 (958.75–2291.31)	0.46 (0.29–0.69)	−3.6 (−4.44--2.76)
Central Europe	1382.59 (696.32–2265.16)	0.96 (0.48–1.57)	885.62 (648.99–1132.93)	0.42 (0.31–0.54)	−2.45 (−2.94--1.96)
Western Europe	2897.67 (1369.3–4861.51)	0.53 (0.25–0.89)	1435.41 (978.03–2032.32)	0.15 (0.1–0.21)	−4.08 (−4.49--3.67)
High-income North America	1163.74 (455.74–2059.58)	0.35 (0.14–0.62)	628.73 (308.36–1026.18)	0.1 (0.05–0.16)	−4.34 (−4.83--3.85)
Andean Latin America	388.37 (188.07–642.09)	1.75 (0.84–2.91)	565.01 (350.29–864)	0.94 (0.58–1.43)	−2.57 (−2.91--2.24)
Central Latin America	631.22 (337.12–1016.29)	0.7 (0.37–1.13)	1169.86 (787.5–1603.93)	0.47 (0.31–0.64)	−1.28 (−1.66--0.9)
Southern Latin America	566.11 (255.04–991.14)	1.24 (0.56–2.17)	396.71 (224.36–613.98)	0.46 (0.26–0.72)	−3.29 (−3.55--3.03)
Tropical Latin America	734 (267.06–1456.55)	0.69 (0.25–1.36)	1134.64 (645.84–1745.82)	0.44 (0.25–0.67)	−1.65 (−2.08--1.22)
Caribbean	138.56 (46.62–272.64)	0.52 (0.18–1.03)	237.75 (120.07–391.44)	0.44 (0.22–0.73)	−0.38 (−0.62--0.15)
North Africa and Middle East	1604.5 (954.99–2567.03)	1.01 (0.58–1.67)	2495.71 (1834.33–3381.13)	0.58 (0.43–0.79)	−1.74 (−1.86--1.63)
Eastern Sub-Saharan Africa	129.64 (36.37–321.32)	0.17 (0.05–0.43)	251.89 (73.01–664.17)	0.14 (0.04–0.38)	−0.27 (−0.42--0.12)
Central Sub-Saharan Africa	43.12 (14.83–98.58)	0.2 (0.07–0.47)	120.54 (41.65–305.2)	0.21 (0.07–0.54)	0.61 (0.41–0.8)
Southern Sub-Saharan Africa	60.56 (38.18–85.53)	0.21 (0.13–0.3)	134.7 (88.85–182.88)	0.23 (0.15–0.31)	0.68 (0.36–1.01)
Western Sub-Saharan Africa	336.54 (109.43–862.02)	0.37 (0.12–0.94)	607.45 (205.17–1469.05)	0.28 (0.1–0.68)	−0.41 (−0.77--0.05)

**Table 2:** Trends in PM_2.5_-attributable subarachnoid hemorrhage DALYs from 1990 to 2021 by geographic region.

Characteristics	1990		2021		1990–2021
	The number of DALYs (95% UI)	Age-standardized DALY rates (95% UI)	The number of DALYs (95% UI)	Age-standardized DALY rates (95% UI)	EAPC of age-standardized DALY rates (95% CI)
Global	1136504.59 (725374.86–1657604.7)	27.12 (17.2–39.78)	1531352.88 (1031890.49–1965982.25)	17.77 (11.98–22.81)	−1.57 (−1.79--1.35)
Female	519297.16 (325552.47–764903.3)	23.83 (14.86–35.11)	701096.65 (460918.33–935483.09)	15.55 (10.23–20.78)	−1.65 (−1.88--1.41)
Male	617207.43 (353967.98–981307.6)	30.68 (17.24–49.23)	830256.23 (549880.1–1108338.4)	20.11 (13.32–26.8)	−1.52 (−1.74--1.29)
Low SDI	29364.8 (11976.45–55548.63)	11.14 (4.48–21.31)	63476.64 (29786.94–119538.2)	10.36 (4.89–19.54)	−0.04 (−0.33–0.25)
Low-middle SDI	114985.65 (60478.91–195482.12)	16.15 (8.38–27.48)	305708.73 (166503.28–471027.81)	19.01 (10.33–29.37)	0.74 (0.48–1)
Middle SDI	453423.98 (241156.84–749808.04)	40.98 (21.3–68.42)	749039.08 (493468.67–974188.99)	27.42 (17.96–35.65)	−1.56 (−1.86--1.26)
High-middle SDI	320158.66 (203251.49–482516.48)	31.54 (20.02–47.6)	286623.85 (208435.32–379811.76)	15.2 (11.06–20.11)	−2.59 (−2.84--2.35)
High SDI	217428.11 (135457.36–319658.05)	20.9 (13.01–30.71)	125494.42 (87242.4–168096.24)	7.14 (5.07–9.5)	−3.7 (−3.87--3.54)
Australasia	1129.61 (38.04–3231.74)	5.01 (0.17–14.33)	1630.4 (964.32–2422.15)	3.51 (2.06–5.21)	−1.62 (−2.14--1.11)
Oceania	494.23 (124.71–1200.94)	13.35 (3.45–33.1)	1204.59 (378.66–2724.32)	12.66 (4.01–28.13)	−0.36 (−0.56--0.16)
East Asia	462152.05 (182873.28–871332.36)	53.42 (20.91–101.51)	563441.41 (351404.57–757793.66)	26.52 (16.55–35.56)	−2.53 (−2.99--2.05)
Central Asia	8619.73 (3429.83–16476.52)	17.08 (6.79–32.69)	20,861 (13714.72–27559.62)	23.71 (15.57–31.27)	1.74 (1.22–2.28)
South Asia	112048.73 (46306.16–221706.98)	16.21 (6.55–32.77)	374251.52 (205343.24–578196.05)	22.75 (12.49–35.31)	1.32 (0.97–1.66)
Southeast Asia	72081.99 (31321.32–135952.62)	24.63 (10.61–46.8)	169509.32 (107009.67–236520.16)	24.16 (15.27–33.78)	−0.49 (−0.72--0.26)
High-income Asia Pacific	64440.63 (18166.53–128105.47)	31.39 (8.89–62.38)	51137.29 (30294.83–75689.97)	14.33 (8.61–21.14)	−2.74 (−3.02--2.46)
Eastern Europe	82583.34 (39862.72–131681.38)	30.86 (14.88–49.18)	40797.18 (25687.4–61238.44)	13.16 (8.32–19.74)	−3.58 (−4.24--2.92)
Central Europe	46349.59 (23110.98–75666.32)	31.99 (15.95–52.18)	23736.47 (17562.96–30334.88)	12.91 (9.55–16.48)	−2.77 (−3.24--2.3)
Western Europe	84976.52 (40828.54–142127.69)	16.89 (8.14–28.3)	32847.55 (22628.83–45727.77)	4.21 (2.89–5.85)	−4.51 (−4.87--4.15)
High-income North America	37555.47 (14752.04–65443.57)	11.72 (4.61–20.42)	16368.8 (8020.23–26548.32)	2.94 (1.44–4.76)	−4.8 (−5.23--4.37)
Andean Latin America	14151.84 (6925.91–22954.87)	57.26 (27.67–93.31)	18350.83 (11429.34–28120.04)	29.14 (18.11–44.62)	−2.75 (−3.09--2.42)
Central Latin America	23833.47 (12728.54–38573.97)	23.21 (12.39–37.58)	36511.78 (24532.9–49058.32)	14.04 (9.42–18.85)	−1.67 (−1.99--1.35)
Southern Latin America	18588.98 (8457.46–32508.09)	39.77 (18.08–69.59)	11829.43 (6783.69–18333.56)	14.44 (8.28–22.36)	−3.44 (−3.67--3.22)
Tropical Latin America	29473.54 (10863.27–58361.13)	25.37 (9.36–50.16)	36069.44 (20845.19–54927.61)	13.69 (7.91–20.84)	−2.31 (−2.7--1.91)
Caribbean	4796.3 (1620.12–9505.47)	17.13 (5.8–34)	7658.72 (3781.95–12670.84)	14.5 (7.17–23.99)	−0.37 (−0.61--0.13)
North Africa and Middle East	52418.13 (32355.21–78672.59)	27.34 (16.56–41.69)	82059.52 (60696.74–107334.87)	15.9 (11.83–20.98)	−1.77 (−1.88--1.66)
Eastern Sub-Saharan Africa	4609.94 (1529.63–10462.43)	5.19 (1.71–11.96)	9572.38 (3238.94–23195.32)	4.43 (1.52–10.72)	−0.13 (−0.28–0.03)
Central Sub-Saharan Africa	1550.14 (601.31–3325.94)	5.89 (2.28–12.88)	4539.92 (1775.84–10751.81)	6.53 (2.58–15.56)	0.74 (0.54–0.94)
Southern Sub-Saharan Africa	2447.72 (1527.92–3438.78)	7.53 (4.7–10.57)	4980.67 (3292.21–6837.76)	7.53 (5.02–10.25)	0.38 (0.1–0.66)
Western Sub-Saharan Africa	12202.66 (4441.79–29598.49)	11.87 (4.33–28.61)	23994.66 (9149.55–53345.06)	9.41 (3.6–20.8)	−0.26 (−0.61–0.11)

**Figure 2:**
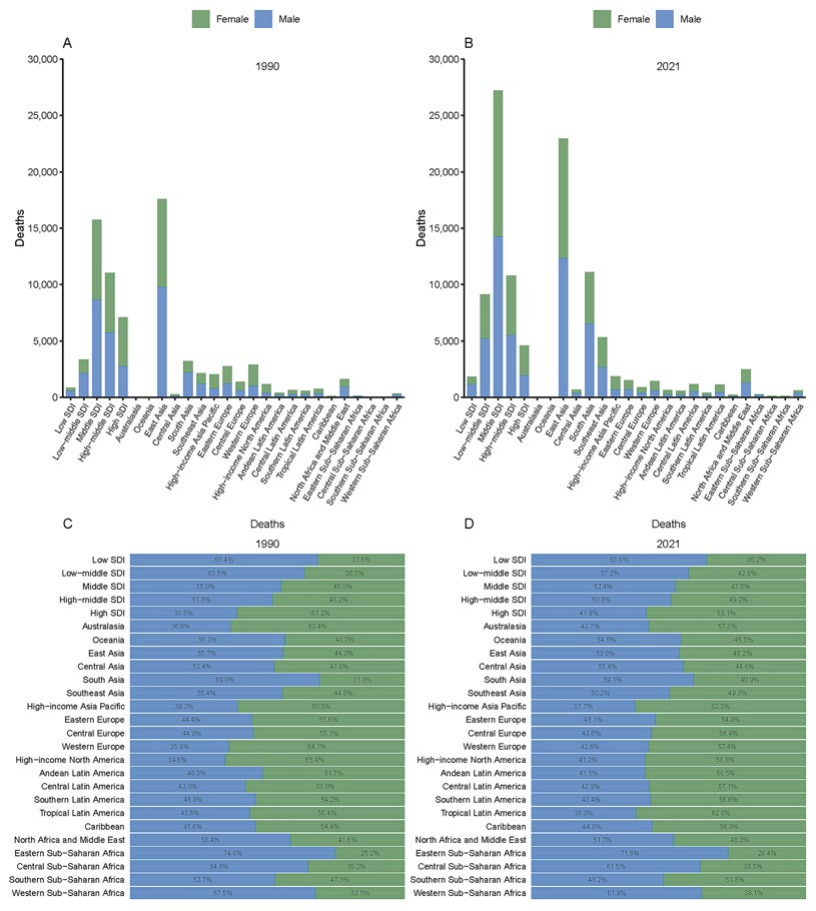
Deaths from subarachnoid hemorrhage attributed to PM2.5 by GBD and SDI regions in 1990 **(A)** and 2021 **(B)**, with proportional distributions in 1990 **(C)** and 2021 **(D)**.

Among the 21 GBD regions, South Asia and Southeast Asia experienced the most notable increase in SAH deaths attributed to PM_2.5_ by 2021 (Figure 2). Death numbers in South and Southeast Asia increased from 3,201.81 and 2,149.93 in 1990 to 11,091.32 and 5,309.19 in 2021. Nevertheless, East Asia bore the greatest burden, with the highest death and DALY figures peaking at 229,552.99 and 563,441.41 (Figure 2, Supplementary Figure S1, and Tables 1, 2). In contrast, high SDI regions such as Australasia and Oceania exhibited lower PM_2.5_-attributable SAH burdens, with death numbers recorded at 66 (95% UI, 39.03–100.57) and 31.82 (95% UI, 9.85–71.45), and DALY numbers at 1,630.4 (95% UI, 964.32–2,422.15) and 1,204.59 (95% UI, 378.66–2,724.32) (Tables 1, 2). Furthermore, in South Asia, the proportion of female deaths increased significantly, from 31.0% in 1990 to 40.9% in 2021 (Figure 2).

### Global trends of PM2.5-related SAH burden

**Figure 3:**
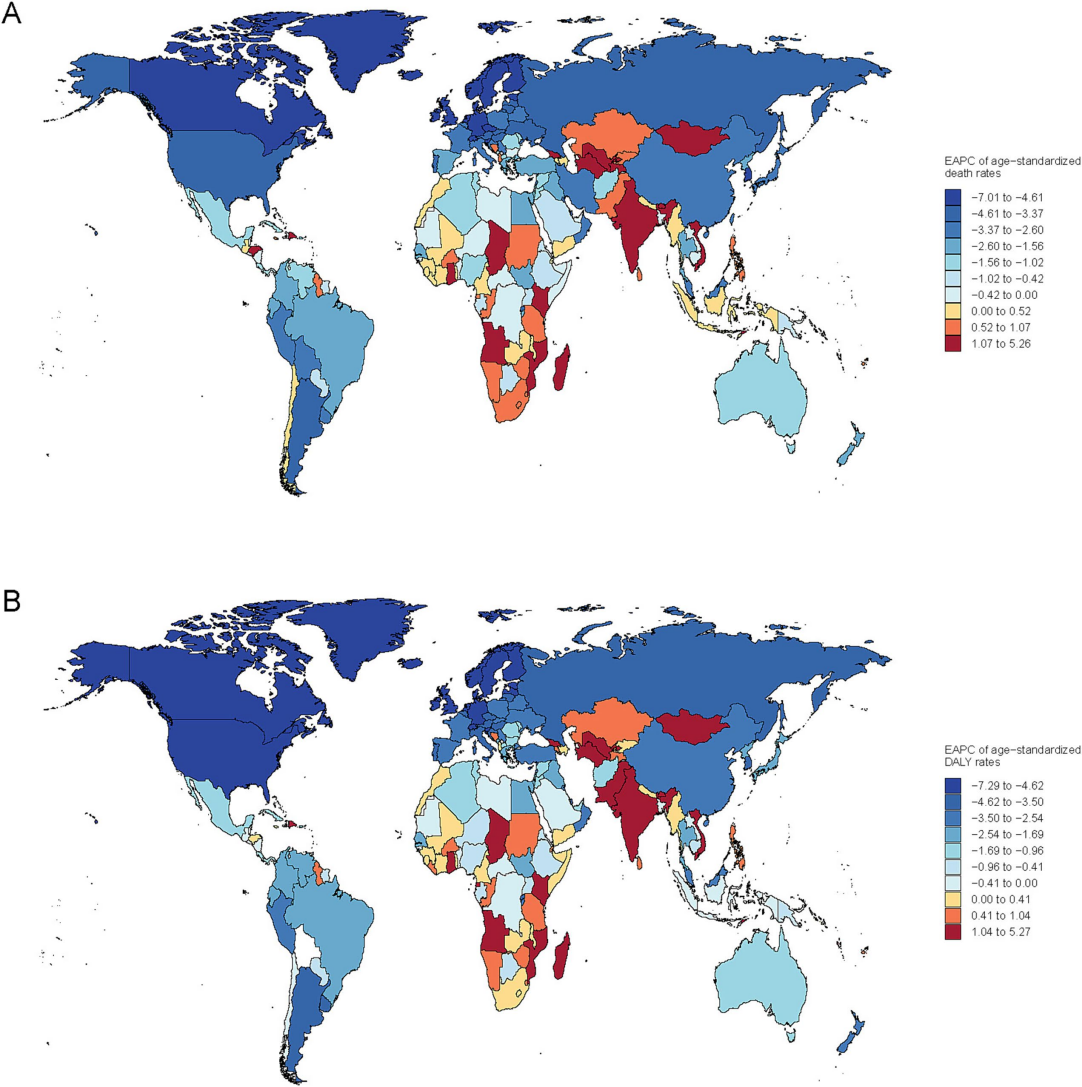
Global map of EAPC for age-standardized rates of subarachnoid hemorrhage due to PM_2.5_ for deaths **(A)** and DALYs **(B)**.

Nationally, Mongolia had the highest burden of subarachnoid hemorrhage attributable to ambient PM_2.5_, with an ASMR of 2.49 (95% UI, 1.23–3.82) and ASDR of 61.92 (95% UI, 30.60–93.24) in 2021. Moreover, the EAPC in ASMR showed the most significant increasing trend at 5.26 (95% CI, 4.69–5.84) (Supplementary Tables S1, S2). Similarly, increasing trends in ASMR and ASDR were noted across South Asia, Central Asia and Southern Sub-Saharan Africa (Figure 3).

Conversely, on a global scale, the EAPC for both ASMR and ASDR showed a decline in most countries, particularly in North America, Europe, Oceania, and South America. Notably, exhibited the most pronounced decreases in EAPC for ASMR and ASDR, at −7.01 (95% CI, −7.48 to −6.53) and −7.25 (95% CI, −7.66 to −6.84), respectively.

### Age-specific global burden of SAH due to PM_2.5_: 1990–2021

**Figure 4:**
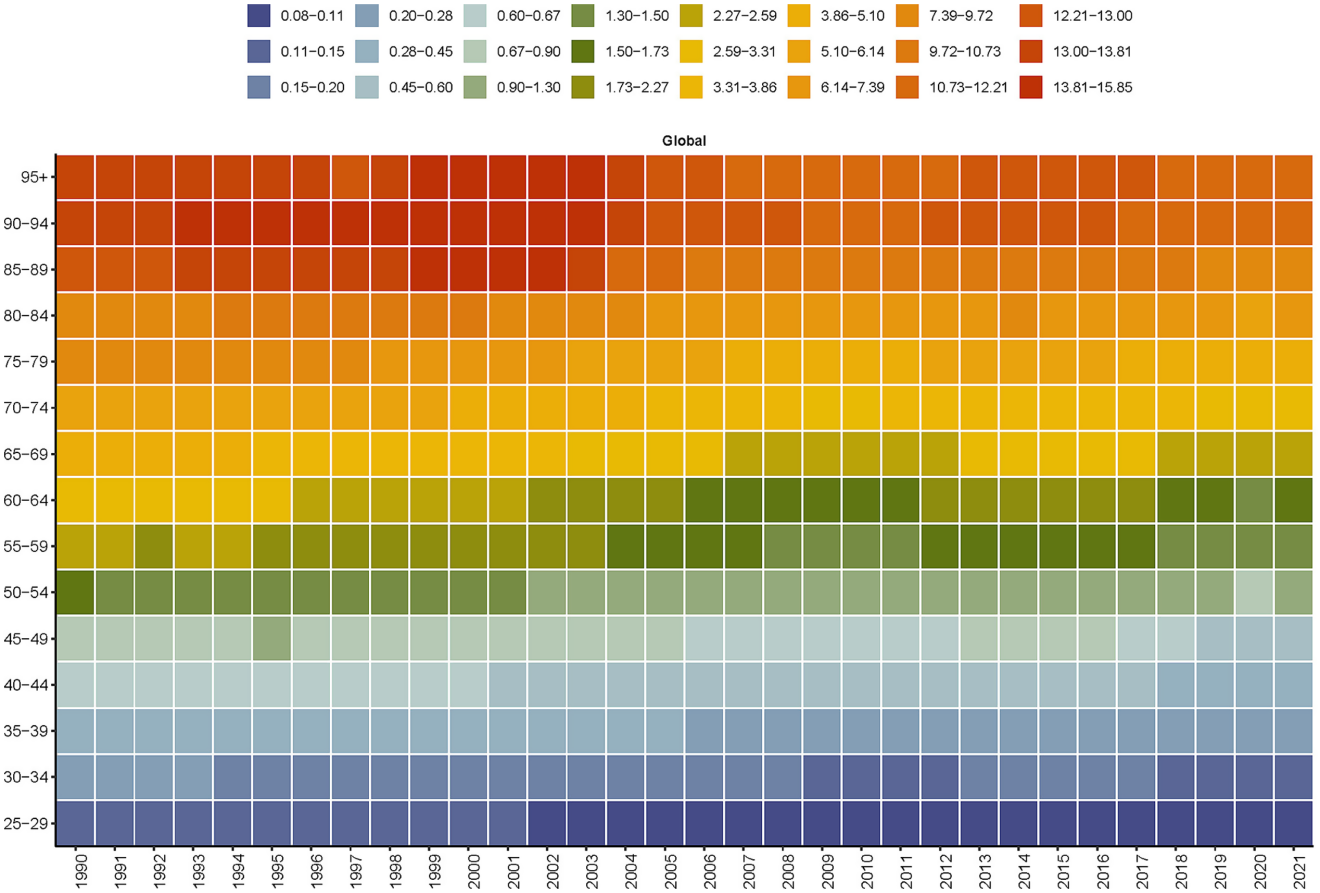
Global age-stratified deaths from subarachnoid hemorrhage attributable to PM_2.5_ from 1990 to 2021.

Between 1990 and 2021, the PM_2.5_-attributable death and DALYs of SAH saw a consistent annual decline across all age groups. By 2021, the disease burden had lessened in comparison to 1990 for every age demographic (Figure 4 and Supplementary Figure S2). Notably, a downward trend in the burden for those over 50 years old was observed starting around 2000. For those under 50, although the death burden was comparatively minor, a decline has been noted since the early 2000s (Figure 4). The death among those aged 85 and above showed a brief increase between 1999 and 2003, followed by a decline. Between 1990 and 2021, there was a notable decrease in global age-stratified DALYs from SAH across all age groups. However, from 2012 to 2017, there was a slight increase in DALYs for individuals aged 70–74 (Supplementary Figure S2).

### PM_2.5_-attributable SAH burden in 2021, SDI-stratified

**Figure 5:**
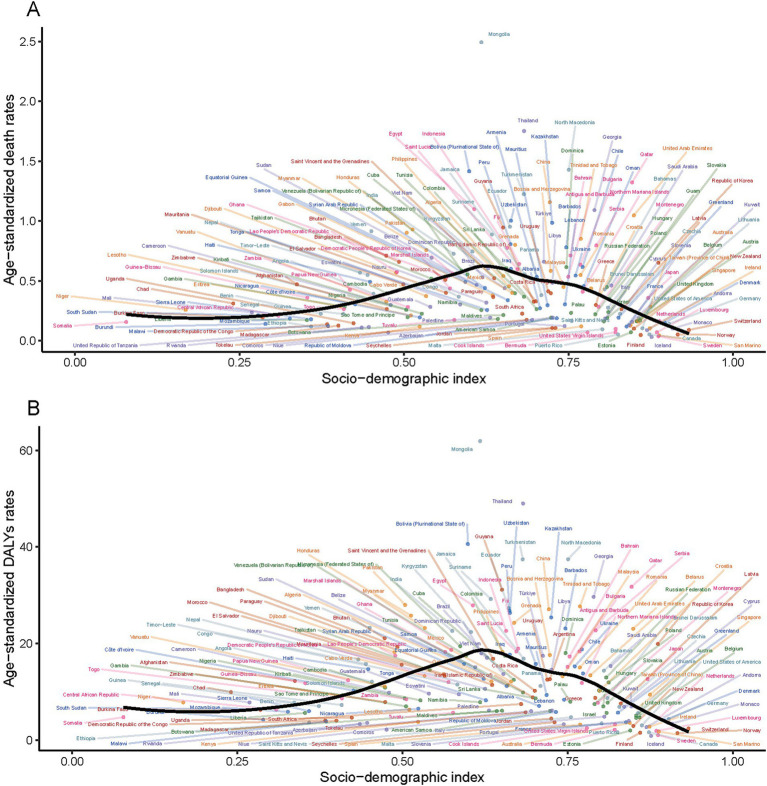
Association between age-standardized rates and the sociodemographic index for subarachnoid hemorrhage due to PM_2.5_ for deaths **(A)** and DALYs **(B)**.

In the comparative analysis of PM_2.5_-attributable SAH burden across countries and territories with varying SDI levels. It was found that the correlation between ASMR and SDI mirrored that between ASDR and SDI. However, a significant trend was not observed, with the *p*-value exceeding 0.05. The disease burden due to PM_2.5_ in SAH was predominantly greater in countries or regions with an SDI ranging from 0.6 to 0.7 and lower in those with an SDI below 0.4 or above 0.8 (Figure 5).

### Future projection of global SAH burden

**Figure 6:**
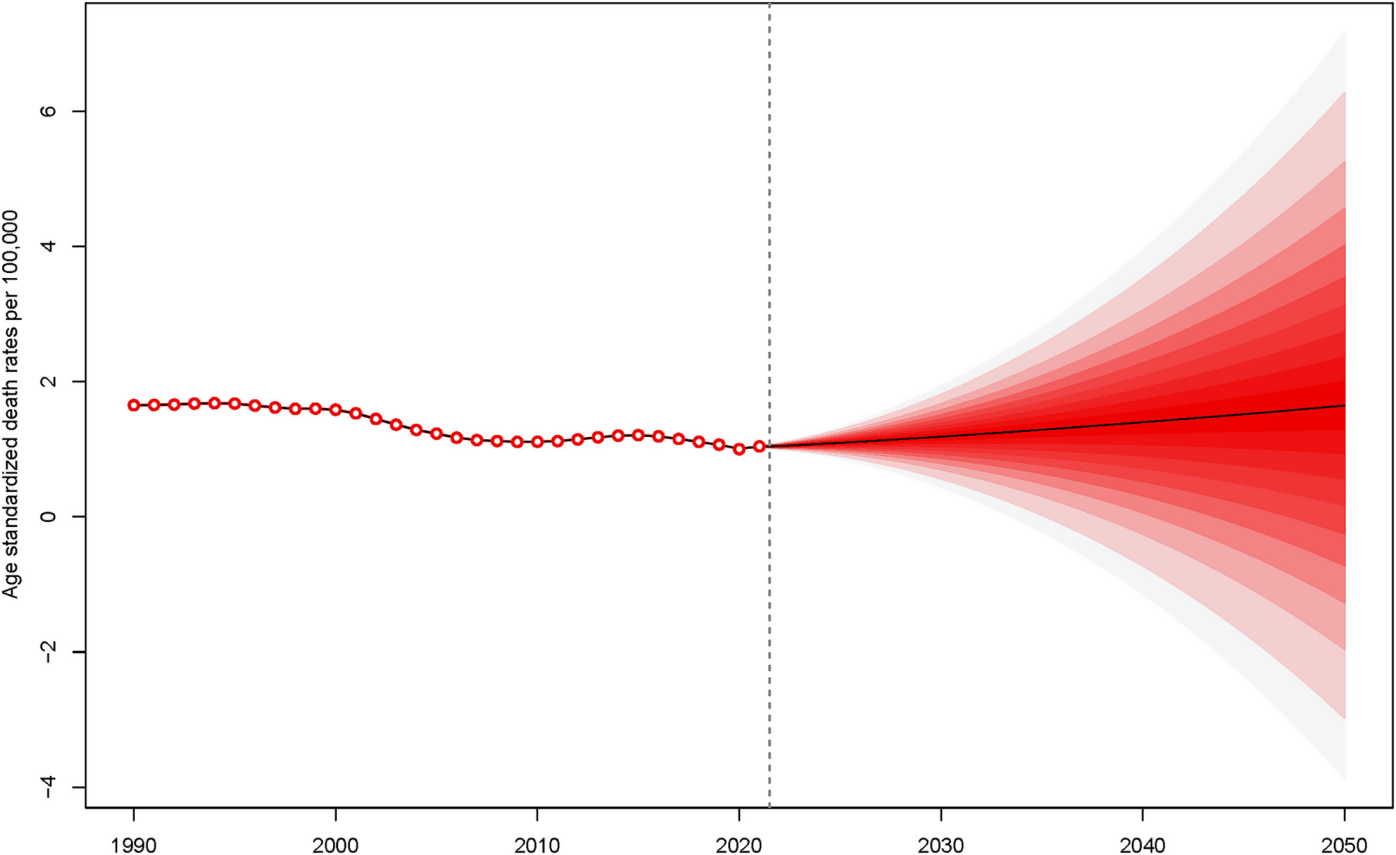
Projection of age-standardized death rates for subarachnoid hemorrhage due to PM_2.5_ from 2022 to 2050.

Between 1990 and 2021, the ASMR and ASDR for SAH attributable to ambient PM_2.5_ exhibited slightly fluctuations. This period witnessed a complex interplay of factors influencing the health outcomes related to ambient PM_2.5_ exposure. Looking ahead, from 2022 to 2050, there is a projected increase in both ASMR and ASDR for SAH due to ambient PM_2.5_, indicating a potential escalation in the disease burden (Figure 6 and Supplementary Figure S3).

## Discussion

### Global trends and paradoxical findings

This study provides a comprehensive analysis of the global disease burden of SAH attributable to ambient PM_2.5_ pollution across regions, genders, age groups, and SDI levels from 1990 to 2021, with projections extending to 2050. While previous studies have investigated PM_2.5_-associated stroke burden, this work uniquely quantifies the sex-specific temporal trends and aging-related vulnerability patterns in SAH burden, while integrating future projections through robust modeling approaches (32–34).

The paradoxical 40.4% increase in absolute SAH deaths from 38,129.84 to 53,561.65 despite a 36.4% decline in age-standardized mortality rates (from 0.99 to 0.63 per 100,000) reflects the complex interplay of demographic and epidemiological factors over the 32-year study period. This apparent contradiction can be attributed to several key demographic transitions: (1) population aging dynamics-the global population aged ≥60 years increased by approximately 180% during this period, creating a substantially larger at-risk population despite improved per-capita risk profiles (35). (2) overall population growth - the world population expanded from 5.3 billion in 1990 to 7.9 billion in 2021, representing a 49% increase in the denominator for absolute case calculations (36). and (3) persistent environmental health disparities - while high-income regions achieved substantial PM_2.5_ reductions, rapid industrialization in South Asia and Sub-Saharan Africa maintained or increased exposure levels for large populations.

This demographic-epidemiologic paradox illustrates a critical limitation in public health assessment: age-standardized rates, while essential for comparing risk across populations and time periods, may underestimate the true societal burden when applied to aging populations with age-sensitive health outcomes. The divergence between these metrics emphasizes that successful risk reduction strategies at the individual level can be overwhelmed by demographic shifts, necessitating integrated approaches that address both exposure reduction and healthcare system capacity for aging populations. Furthermore, the absolute increase in deaths predominantly occurred in low-and middle-income countries (contributing 78% of the excess deaths), highlighting persistent global health inequities in environmental protection and healthcare access (37, 38).

### Regional disparities and socioeconomic development index patterns

The pronounced regional disparities underscore differential progress in environmental health. High SDI regions achieved the most substantial improvements (ASMR: 0.66 to 0.22), likely reflecting stringent air quality standards and advanced healthcare systems (39). The 65.8% ASDR reduction in high SDI regions (20.9 to 7.14) demonstrates the effectiveness of integrated environmental-health interventions, providing a roadmap for middle SDI countries (40).

Conversely, middle SDI regions bear the highest SAH burden (ASMR 1.07), trapped in a developmental phase combining industrializing economies with insufficient pollution controls. These regions experience rapid industrialization, environmental pollution, and limited medical resources, leading to a higher disease burden of PM_2.5_-related SAH, particularly in South and East Asia (41). Low-middle SDI regions’ unfavorable trends may indicate persistent barriers in pollution control and healthcare access (42).

### Geographical distribution and country-specific findings

The striking geographical disparities in subarachnoid hemorrhage burden attributable to ambient PM_2.5_ exposure reflect the complex interplay between environmental pollution levels, healthcare infrastructure, and socioeconomic development across different regions. Mongolia’s position as having the highest national burden, with an age-standardized mortality rate of 2.49 per 100,000 and a concerning annual increase of 5.26%, underscores the urgent need for targeted air quality interventions in this region.

South and Southeast Asia exhibited the steepest increases in PM_2.5_-attributable SAH deaths (South Asia: +246.5%, Southeast Asia: +147.0%), aligning with satellite-derived PM_2.5_ concentration trends showing population-weighted annual averages exceeding 75 μg/m^3^ in Bangladesh and India (43). These regions face compounded challenges including inadequate neurosurgical infrastructure (44) and limited implementation of WHO air quality guidelines (45, 46) creating a “double burden” of environmental and healthcare system deficiencies. The pronounced increasing trends observed in South Asia, Central Asia, and Southern Sub-Saharan Africa align with the rapid industrialization and urbanization occurring (Figure 3) (47, 48). Air pollution in South Asia results from a complex interplay of emission sources beyond industrial activities, including the combustion of solid fuels for cooking and heating, emissions from small industries such as brick kilns, the burning of municipal and agricultural waste, and cremation practices (49, 50). In contrast, East Asia’s substantial absolute burden (229,552.99 deaths in 2021) reflects legacy pollution effects from rapid industrialization, though recent policy interventions show promising declines in PM_2.5_ levels (51). The declining trends in North America, Europe, Oceania, and South America reflect successful implementation of air quality improvement policies and stricter environmental regulations over the past decades, demonstrating the potential for effective public health interventions to reduce PM_2.5_-related health burdens (36, 40). East Asia region includes China, North Korea, and Taiwan, with China contributing the vast majority of the 229,553 deaths in 2021 due to its large population size. Despite China’s middle SDI classification, this region showed significant improvements in age-standardized rates (ASMR decline), reflecting substantial investments in healthcare infrastructure and air quality management over the study period (52). The apparent contradiction between high absolute burden and improving rates reflects China’s demographic transition and successful implementation of pollution control policies since 2013 (53). High-income Asia-Pacific region encompasses Australia, Brunei, Japan, New Zealand, Singapore, and South Korea. These countries consistently demonstrate the lowest PM_2.5_-attributable SAH burden, with marked improvements in both absolute and age-standardized metrics, directly correlating with their high SDI status and advanced healthcare systems (48). The steep declining trends in this region exemplify how combined high socioeconomic development and stringent environmental regulations effectively reduce pollution-related health burdens.

### Sex and age dimensions

The male predominance in PM_2.5_-related SAH mortality (ASMR 0.72 vs. 0.55 in females) likely reflects differential exposure patterns and biological susceptibility. Men experience higher occupational exposure through industrial work, while sex-linked differences in inflammatory responses and hormonal status may influence cardiovascular vulnerability to PM_2.5_ (54, 55). However, evidence on gender differences in air pollution health effects remains inconsistent across studies (56).

The global increase in female SAH mortality proportion (particularly +9.9% in South Asia) may be explained through two complementary mechanisms: biological susceptibility via enhanced oxidative stress responses (20, 21), and socioeconomic factors limiting women’s access to preventive healthcare in LMICs (57). The age-dependent burden escalation (Figure 4 and Supplementary Figure S2) demonstrates a 3.2-fold higher mortality risk in populations >70 years compared to <50 years, likely mediated through PM_2.5_-induced exacerbation of hypertension (58) and cumulative blood–brain barrier damage (16)^.^ This aging-related vulnerability is projected to intensify as the global population over 60 years grows by 56% by 2050 (23).

### Future projections and public health implications

The increased burden of SAH projected by the model over the next 29 years (Figure 6 and Supplementary Figure S1), potentially due to a significant association between PM_2.5_ exposure and SAH, with a particularly sharp increase in risk over short periods. As the global population ages, the older adults, who are more sensitive to the health effects of PM_2.5_, become a larger proportion of society (59). Additionally, industrialization and urbanization have exacerbated PM_2.5_ pollution, and regions lacking effective health protection measures and resources experience more significant adverse health effects from PM_2.5_ (41, 60). Therefore, it is imperative that the world invests more time and effort into controlling PM_2.5_ pollution and reducing the medical burden on SAH patients (61).

Previous research has examined the global burden of PM_2.5_ in relation to SAH, yet this study extends these findings, revealing that between 1990 and 2021, the proportion of SAH attributed to PM_2.5_ increased among women across all GBD regions. This underscores a concerning trend, particularly as the disease burden of SAH due to PM_2.5_ escalating with advancing age. Our projections for future trends indicate a global increase in both SAH deaths and DALYs attributed to PM_2.5_.

Since the database used in this study covers different countries and regions, cultural, geographical, climatic, and even genetic differences have influenced the study’s results. Although these factors are unlikely to fundamentally alter the relationship between ambient air pollution and the risk of SAH (62, 63). Genetic polymorphisms in oxidative stress pathways and inflammatory responses may modulate individual susceptibility to PM_2.5_-induced cerebrovascular damage. This could potentially explain some of the striking regional differences, such as East Asia having a disproportionately high absolute burden despite relatively lower PM_2.5_ concentrations (64, 65). Climatic factors, including temperature extremes and seasonal variations, may interact with PM_2.5_ toxicity through altered particulate composition and enhanced inflammatory responses. This could partially account for Mongolia’s exceptionally high burden and the seasonal patterns observed in temperate regions (66). However, the consistent dose–response relationship between PM_2.5_ exposure and SAH risk documented across diverse populations in epidemiological studies suggests that ambient particulate matter remains an independent risk factor regardless of these population-specific modifiers (67).

While the GBD 2021 methodology employs comparative risk assessment that inherently adjusts for major confounders through integrated exposure-response functions derived from epidemiological meta-analyses, residual confounding from traditional SAH risk factors may influence our estimates (68, 69). Chronic diseases (hypertension, diabetes), lifestyle factors (smoking, alcohol consumption), and healthcare access disparities may cluster geographically with PM_2.5_ exposure patterns, potentially inflating the pollution-attributable burden in regions with limited diagnostic capacity and neurosurgical infrastructure (70). The stark regional disparities observed—particularly Mongolia’s exceptionally high burden and South Asia’s 246% mortality growth—likely reflect complex interactions between air pollution exposure and unmeasured socioeconomic, healthcare, and behavioral confounders that extend beyond PM_2.5_ effects alone (71). Despite these limitations, the consistent global patterns and projected 2050 increases suggest that PM_2.5_ reduction efforts would yield substantial health benefits, warranting urgent air quality interventions in high-burden regions regardless of residual confounding concerns.

Our study had some limitations including: (1) Data heterogeneity in cause-of-death certification and PM_2.5_ exposure modeling across regions may affect burden estimates; (2) Residual confounding (e.g., unmeasured comorbidities, socioeconomic factors) could bias risk associations; (3) Limited granularity in occupational/lifestyle exposure data hinders precise gender-risk stratification; (4) Model uncertainties (e.g., counterfactual exposure thresholds) warrant sensitivity analyses in future work. Future there are urgent actions remain imperative: Prioritize air quality interventions in high-burden regions (Mongolia/South/Southeast Asia); Strengthen stroke care systems in low-resource settings; Implement gender-responsive strategies addressing occupational (male) and household (female) exposures; Target hypertension control in older populations. Integrating SAH burden metrics into global health frameworks is essential to mitigate this preventable crisis, despite current methodological constraints.

## Conclusion

This study uncovers a critical paradox in PM_2.5_-attributable subarachnoid hemorrhage (SAH): despite a 36.4% global decline in age-standardized mortality (1990–2021), absolute deaths surged by 40.4%-driven by population aging and growth. Stark inequities persist, with South Asia experiencing a 246.5% death increase and Mongolia bearing the highest burden (ASMR 2.49). Projections indicate rising rates by 2050, disproportionately affecting aging populations and regions with weak pollution controls. Urgent actions are warranted: (1) Prioritize air quality interventions in high-burden regions (Mongolia/South/Southeast Asia); (2) Strengthen stroke care systems in low-resource settings; (3) Implement gender-responsive strategies addressing occupational (male) and household (female) exposures; (4) Target hypertension control in older populations. Integrating SAH burden metrics into global health frameworks is essential to mitigate this preventable crisis.

## Data Availability

The original contributions presented in the study are included in the article/Supplementary material, further inquiries can be directed to the corresponding authors.

## Supplementary material

SUPPLEMENTARY FIGURE S1 DALYs from subarachnoid hemorrhage attributed to PM_2.5_ by GBD and SDI regions in 1990 **(A)** and 2021 **(B)**, with proportional distributions in 1990 **(C)** and 2021 **(D)**.

SUPPLEMENTARY FIGURE S2 Global age-stratified DALYs from subarachnoid hemorrhage attributable to PM_2.5_ from 1990 to 2021.

SUPPLEMENTARY FIGURE S3 Projection of age-standardized DALY rates for subarachnoid hemorrhage due to PM_2.5_ from 2022 to 2050.
