# Supplementary figures and images for: Global burden of subarachnoid hemorrhage attributable to ambient PM2.5 in low-resource regions (1990–2050)
Source: Front Public Health. 2025 Sep 23;13:1652872. doi: 10.3389/fpubh.2025.1652872 (PMC12500674; doi:10.3389/fpubh.2025.1652872)

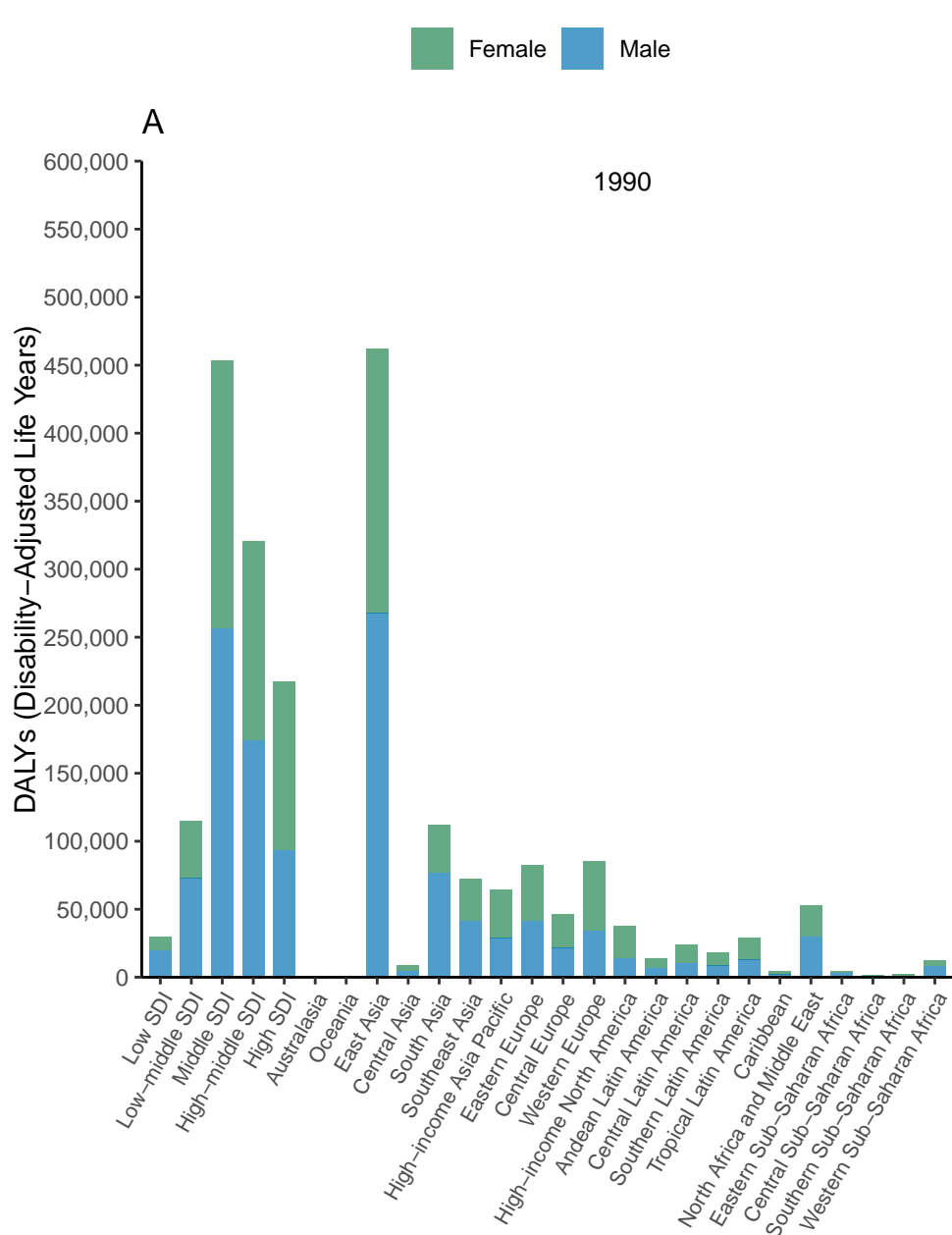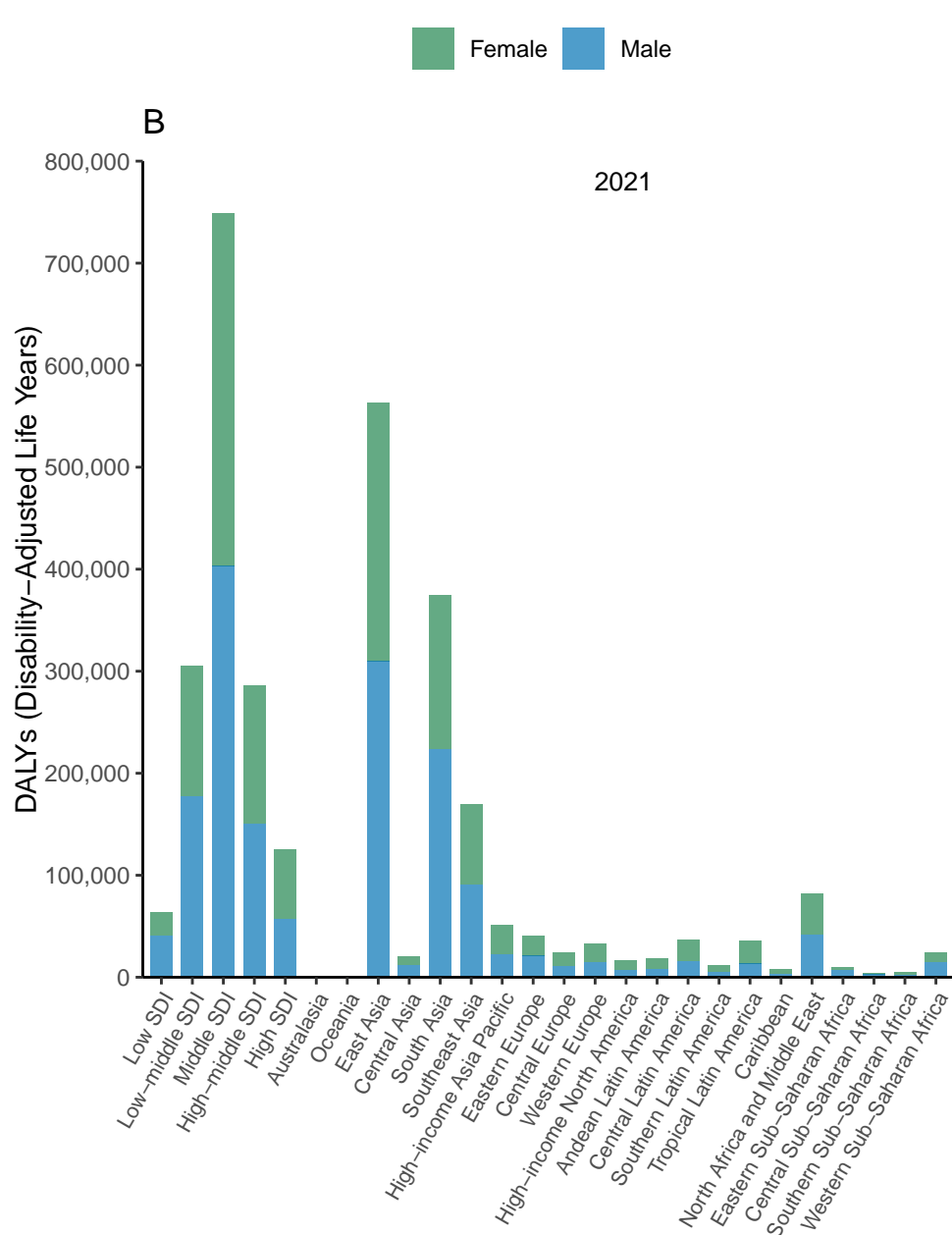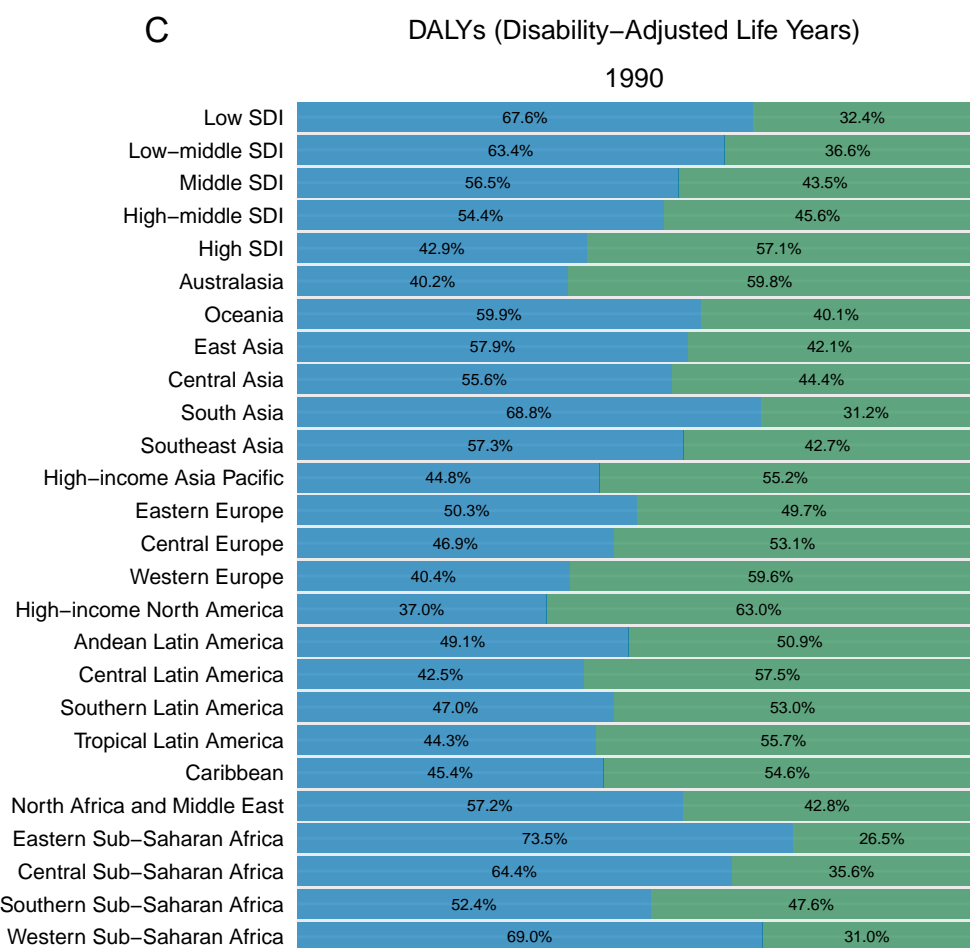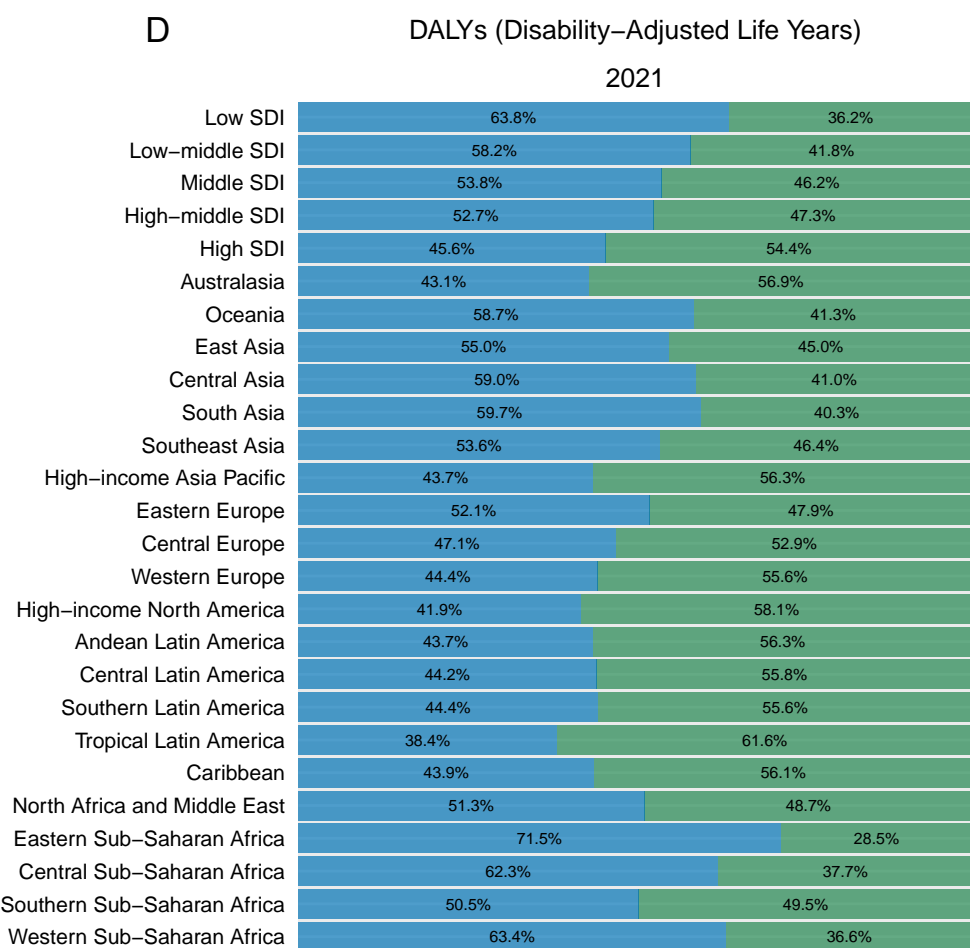

Supplement: SUPPLEMENTARY FIGURE S1 — DALYs from subarachnoid hemorrhage attributed to PM2.5 by GBD and SDI regions in 1990 (A) and 2021 (B), with proportional distributions in 1990 (C) and 2021 (D). [file Data_Sheet_1.PDF]

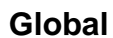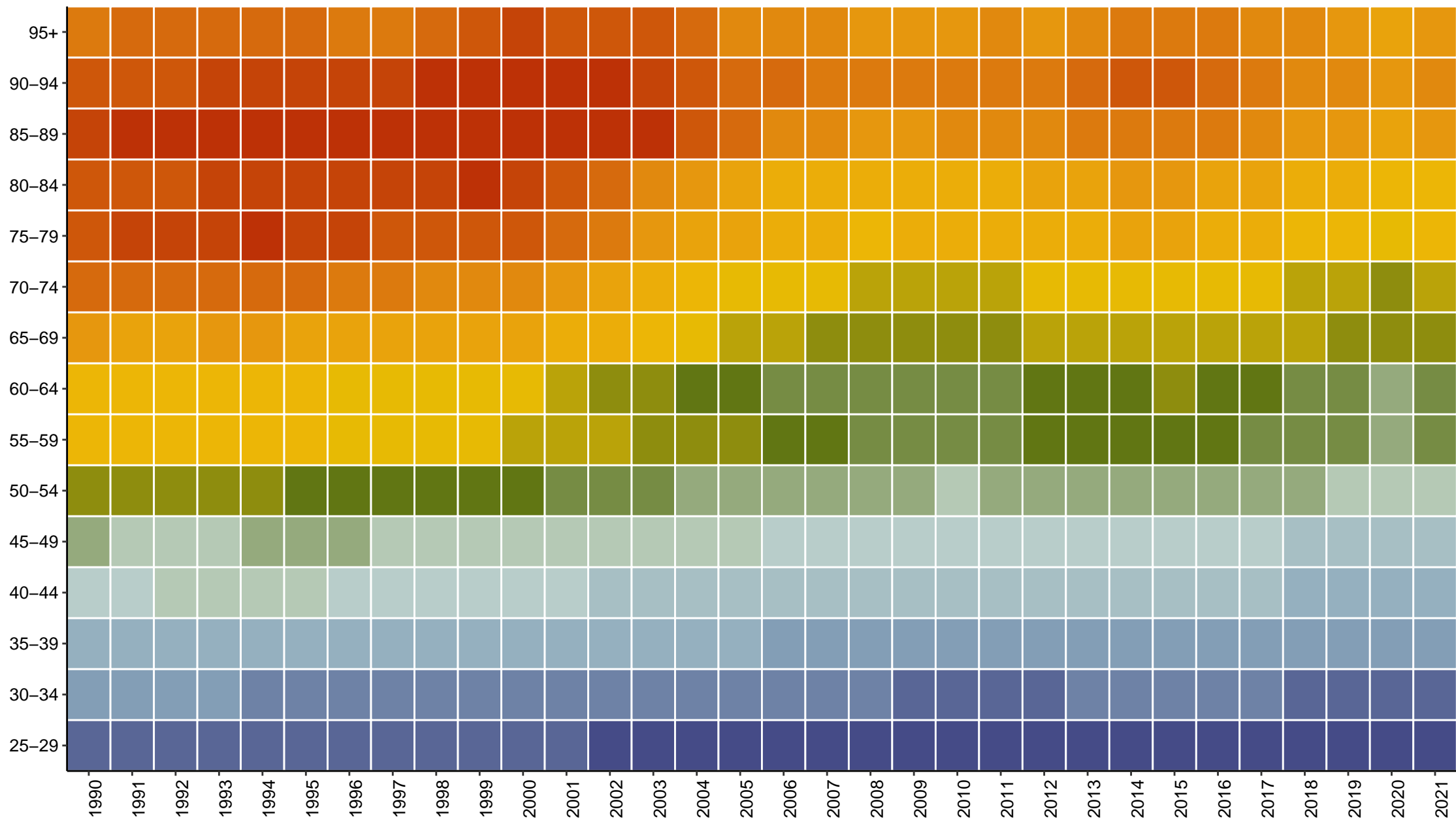

Supplement: SUPPLEMENTARY FIGURE S2 — Global age-stratified DALYs from subarachnoid hemorrhage attributable to PM2.5 from 1990 to 2021. [file Data_Sheet_2.PDF]

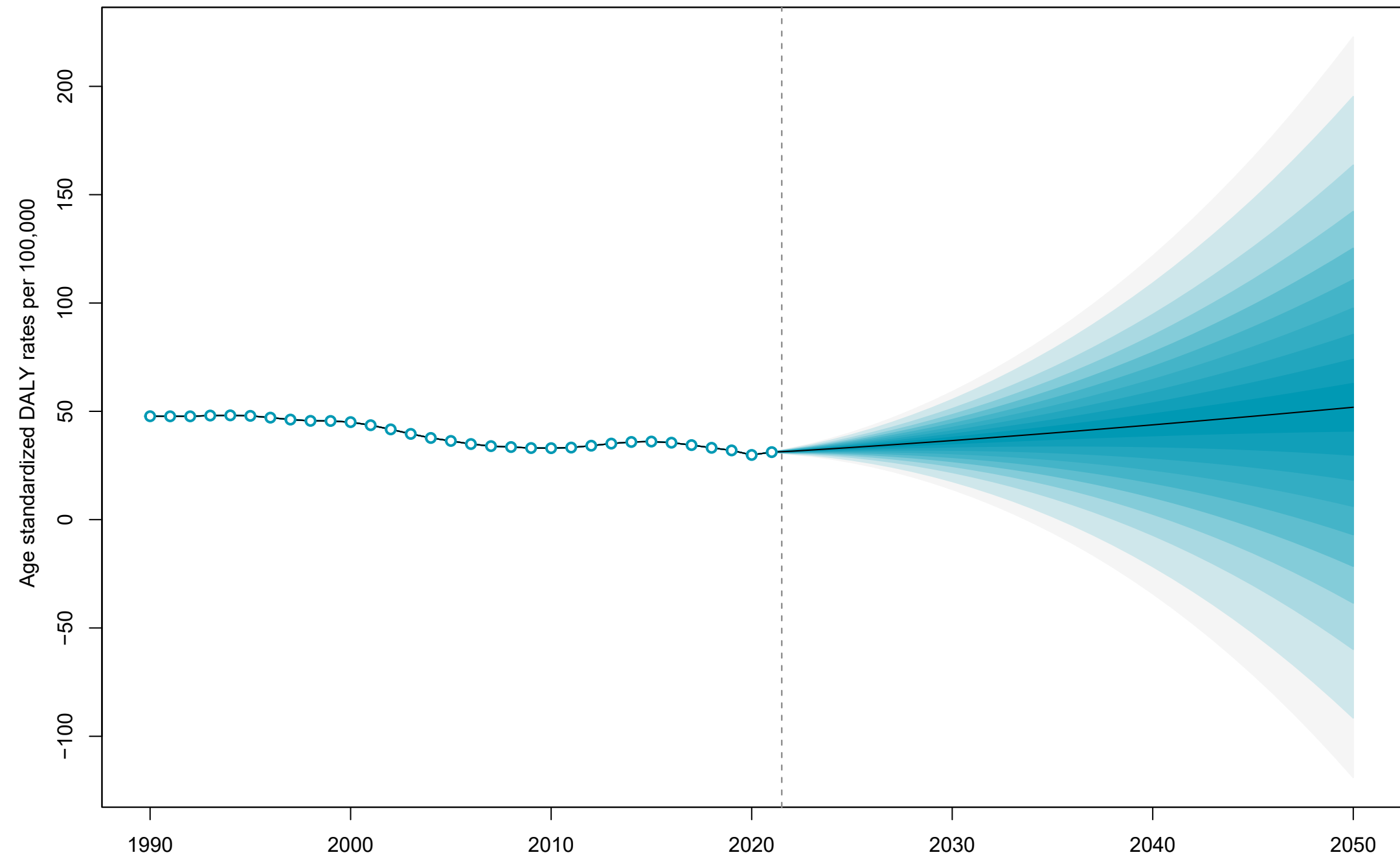

Supplement: SUPPLEMENTARY FIGURE S3 — Projection of age-standardized DALY rates for subarachnoid hemorrhage due to PM2.5 from 2022 to 2050. [file Data_Sheet_3.PDF]
